# Combining Electromagnetic Spectroscopy and Ground-Penetrating Radar for the Detection of Anti-Personnel Landmines

**DOI:** 10.3390/s19153390

**Published:** 2019-08-02

**Authors:** Liam A. Marsh, Wouter van Verre, John L. Davidson, Xianyang Gao, Frank J. W. Podd, David J. Daniels, Anthony J. Peyton

**Affiliations:** The Department of Electrical and Electronic Engineering, The University of Manchester, Oxford Road, Manchester M13 9PL, UK

**Keywords:** electromagnetic induction, magnetic spectroscopy, ground-penetrating radar, multi-modal sensing, landmine detection, explosive remnants of war, metal characterisation

## Abstract

Dual mode detectors combining metal detection and ground-penetrating radar are increasingly being used during humanitarian demining operations because of their ability to discriminate metal clutter. There are many reports in the academic literature studying metal detector and ground-penetrating radar systems individually. However, the combination of these techniques has received much less attention. This paper describes the development of a novel dual modality landmine detector, which integrates spectroscopic metal detection with ground-penetrating radar. This paper presents a feature-level sensor fusion strategy based on three features extracted from the two sensors. This paper shows how the data from the two components can be fused together to enrich the feedback to the operator. The algorithms presented in this paper are targeted at automating the location of buried, visibly obscured objects; however, the system described is also capable of collecting information which could also be used for the potential classification of such items.

## 1. Introduction

In 2017, the latest year for which statistics are available, there were at least 7239 recorded casualties due to landmines and other forms of explosive remnants of war (ERW) in 53 areas and states around the world [1]. Casualties due to the landmines and ERW saw a sharp rise in 2015, and have stayed at a high level since then, with the vast majority (87%) of the victims being civilians. Children are disproportionately represented amongst the reported casualty figures, representing 47% of all civilian casualties for whom the age was known in 2017 [1]. These figures underline the scale of the humanitarian demining problem and the need for urgent action to address it.

Manual demining involves the careful identification and removal of all targets in the mine affected area, and is typically carried out in accordance with the *Standard Operating Procedures* as defined by the UN [2]. This procedure may take a variety of forms, typically involving a team of human operators equipped with metal detectors and ground prodders [3], although other methods such as the deployment of specially trained explosive detection dogs and their handlers are also used [4]. The process of manual detection and excavation is usually very slow, potentially dangerous and costly. The removal cost per mine is estimated to be 300–1000 USD [5], compared with an estimated mine production cost of 3–30 USD [6]. This manual demining clearance rate is unlikely to improve without a significant change in the technology available to the deminers.

**Figure 1:**
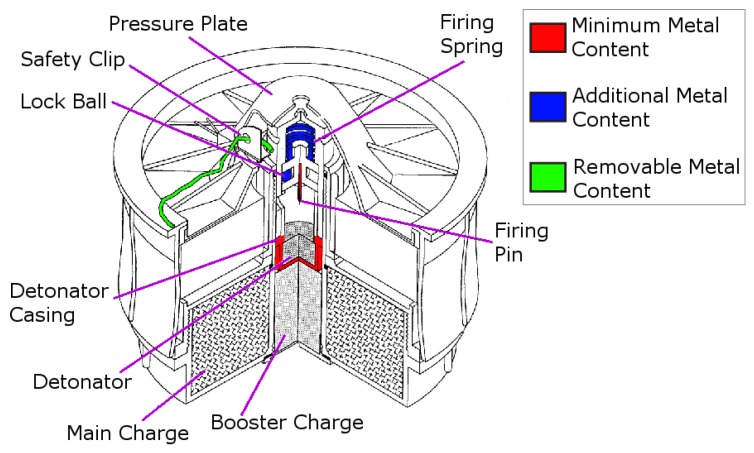
Metallic components present in minimum-metal anti-personnel mines. Adapted from [9].

Inductive metal detection (MD) technology is at the forefront of landmine detection and has been for several decades [7]. However, modern AP landmines typically contain only a few grams of metal [8], making them challenging targets for traditional metal detectors. Figure 1 indicates the parts of a minimum metal landmine (an R2M2 in this case) that contain metal components [9]. Minimum metal landmines often only include the components shown in red, although some may also include the components shown in blue. AP landmines can still be dangerous when buried as deep as 15–20 cm [10].

To detect these small metal components, metal detectors must be extremely sensitive, which presents a problem when there is a significant amount of innocuous metallic clutter, such as shrapnel, also present in the ground, or when the ground is uncooperative due to magnetic mineral content. Using current technology, a deminer must carefully excavate every positive indication, treating it as a potential threat. According to a report from the UN [11], there are 100–300 recorded innocuous metallic items excavated for every landmine. Magnetic Induction Spectroscopy (MIS) offers the potential to overcome many of the problems caused by metallic clutter by matching distinct spectral signatures of legitimate threats rather than looking for a general perturbation as is often the case with traditional MD systems. To further reduce the false alarm rate (FAR) it is desirable to combine metal detection/characterisation technology with other sensing modalities, such as ground-penetrating radar (GPR). Using a GPR to verify the MD response has been shown to significantly reduce the FAR [12,13,14,15].

GPR detects the energy reflected when incident transmitted energy encounters an impedance discontinuity and is backscattered. This impedance inhomogeneity can be the conductivity contrast between a metal mine and the surrounding soil, or the permittivity contrast between a minimum-metal mine and the soil [16]. GPR offers the advantage over metal detectors that it can be used to detect the non-metallic components of an AP landmine. Given the volume of a landmine that is invisible to metal detectors, as shown in Figure 1, GPR should aid the detection of metal free and minimum metal mines.

Although there are many examples of standalone metal detectors, standalone GPR detectors for AP mine detection are less common. This may be because GPR systems are typically more susceptible to clutter in the form of natural discontinuities in the ground conditions such as rocks, tree roots and soil inhomogeneity and perhaps also due to an understandable reluctance to change to a new technology. GPR systems are typically used to augment metal detector systems, with the metal detector forming the primary detection method. In this mode of operation, the GPR is normally used following a positive metal detection signal, as a means of discriminating between a lone scrap metal item and a metal item with impedance discontinuities surrounding it [8]. Hand-held systems that have been developed to date include the US Army’s HSTAMIDS [17], developed by Cyterra (US); the MINEHOUND [18] system manufactured by Vallon (Germany) in partnership with Cobham (UK); and ALIS [19] developed by CEIA (Italy) and Tohoku University (Japan).

Combining GPR systems with metal detectors has been shown to reduce the rate of false alarms, and over the years the clutter rejection rate has improved. For the ALIS system, it was found that having the GPR verify the MDbreduced the FAR by approximately 50%, as reported in 2009 [20]. By 2018, the rejection rate reported for the ALIS system had been improved to 77% [15]. Commercial systems report even higher rejection rates with MINEHOUND reporting an average rejection rate of 92% over a three-year trial in Cambodia, with the initial rejection rate improving from 78% in 2010 to 95% in 2013 [13]. The other commercially available system, HSTAMIDS, was trialled by The HALO Trust in Angola, who reported a rejection rate of 96.5% [14].

The paper describes the development of a dual modality landmine detector, which integrates spectroscopic metal detection with GPR. The paper also presents a feature-level sensor fusion strategy based on three features extracted from the two sensors. A similar method has previously been published by researchers at Duke University (USA) [21]. This paper shows how the data from the two components can be fused together to enrich the feedback to the operator.

The paper is structured as follows. Section 2 contains a description of the prototype system evaluated here. Section 3 describes the measurement methodology adopted for this experiment. The results are discussed in Section 4, which also contains two examples on how the dual-modality sensor enhances the feedback that can be given to the operator. Finally, Section 5 contains the concluding remarks.

## 2. System Overview

### 2.1. Spectroscopic Metal Detection

Inductive metal detectors operate by applying an AC magnetic field to a defined region of space and subsequently measuring the change in that field as a result of any metallic objects within the vicinity of the sensor. Such systems generally apply thresholding techniques to trigger an alarm above a certain limit of perturbation. This method of metal detection is applied to a variety of fields such as aviation security, food safety and recycling. However, the technique is often only partially selective to the recognition of a particular metal object, with the selection method based on metrics such as the phase of the object’s in-phase and quadrature response at a given frequency [22], or by library-fitting broadband time-domain responses of known objects [23,24,25].

**Figure 2:**
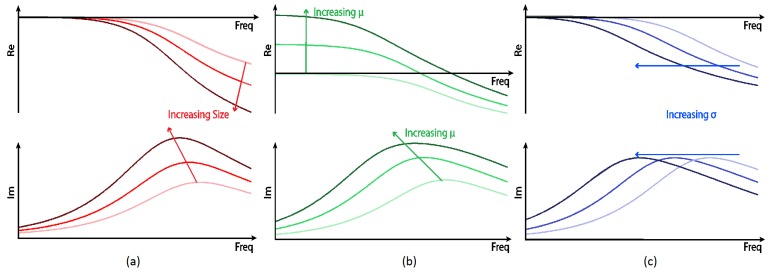
Typical complex spectral responses for: (**a**) increasing object size; (**b**) increasing object permeability μ; and (**c**) increasing object conductivity σ © 2016 IEEE [27].

Metallic objects are known to respond to magnetic fields in a complex manner as a function of their conductivity, permeability, size, and the frequency of the applied field. Figure 2 shows the nature of these dependencies, where the underlying theory behind these spectroscopic trends has been reported in [26]. Through the implementation of MIS, the sensor used in this study can exploit a distinctive spectral response to characterise metallic targets. This spectral response offers the potential to reduce the FAR of the sensor either by rejecting obvious clutter items, e.g., ferrous or extremely large items, or by matching the detector response with pre-recorded spectral responses of known mine targets. This latter approach is described in more detail in our previous work [27,28], and has also been reported in [29]. A similar approach has been used by Geophex (USA) with their GEM-3 sensor [30], which also implements a form of MIS. Examples of classification of landmines from MIS measurements have also previously been reported by the Georgia Institute of Technology (USA) [31,32], and the University of Missouri and the University of Florida (USA) [33]. The MIS system used in this paper has several unique features. This includes a high data output rate (60 Hz); dynamic range extension of the receive electronics through electronic compensation of the primary field; and a large number of allowable simultaneous excitation frequencies (32 harmonics). Some of these features are discussed in more detail in this section. The system presented in this paper also benefits from the advantage of data fusion with the GPR system for enhanced detection capabilities.

**Figure 3:**
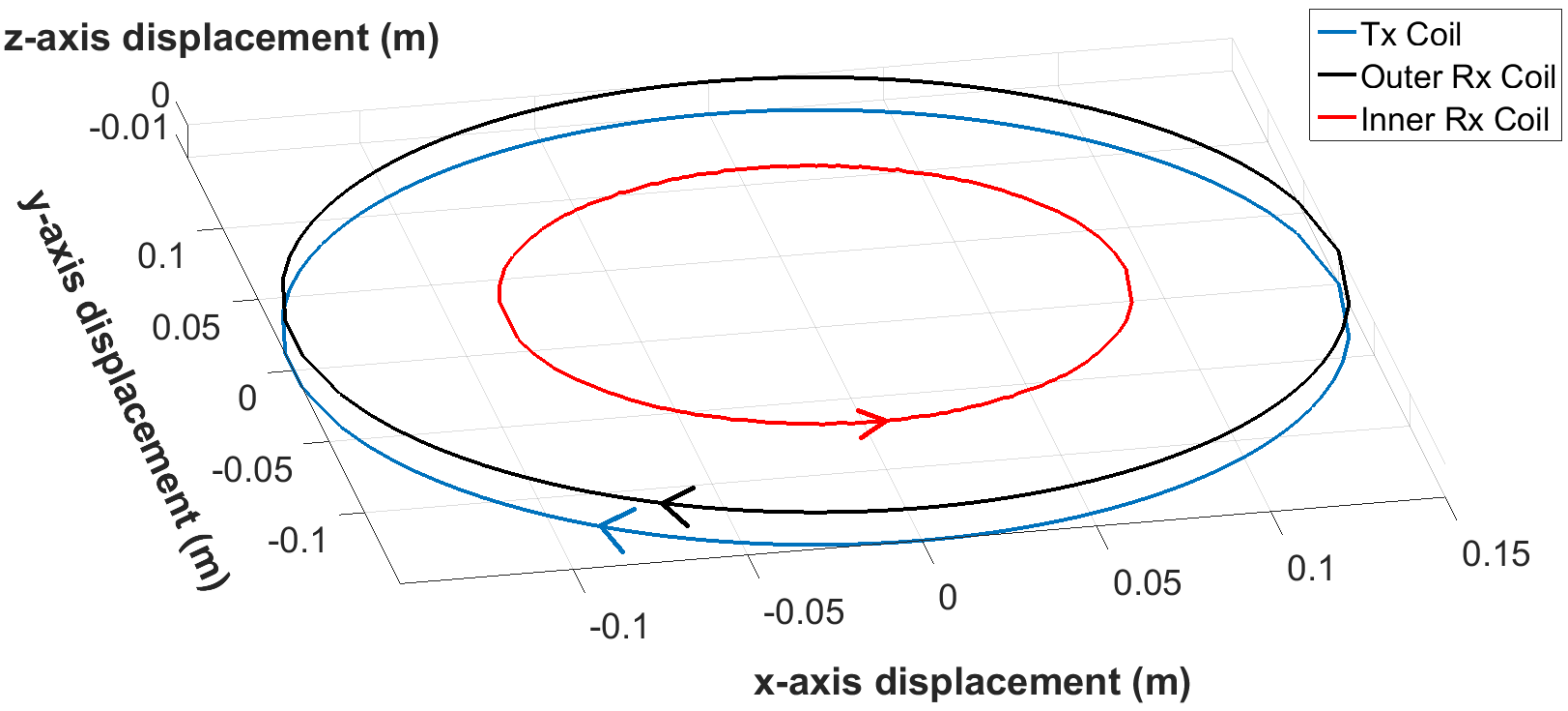
MIS sensor coil geometry including winding directions.

The MIS measurement system is capable of measuring transimpedance values from a pair of coils. The coil dimensions are shown in Figure 3. The transmit coil produces a multi-frequency magnetic field, and the receive coil is used to detect the voltage induced by the combined response of the applied primary field (i.e., that field that would exist without the presence of the object), and any scattering or secondary fields produced as a result of the presence of an object. The receive coil consists of an inner and an outer winding which are connected in series opposition with an appropriate number of turns to ensure near-zero net induced voltage. The values of transimpedance are calculated by dividing the relevant complex voltage harmonics induced in the receive coil by the corresponding complex current harmonics in the transmit coil.

The detector can also employ dynamic ground compensation whereby an active multi-frequency compensation signal is produced and injected into the MIS signal path to null the unwanted signal caused by the electrical and magnetic properties of the ground, thereby giving a near-zero resultant signature. However, even with dynamic ground compensation, the system remains sensitive to inhomogeneities in the soil content, and these can cause clutter in the signal.

Table 1 shows the distribution of transmit harmonics used by the MIS system. These transmit harmonics have been weighted with a $\frac{1}{\sqrt{f}}$ relationship, except the lowest frequency. This first harmonic does not follow this relationship as to do so would apply a large weighting to this frequency component; this would detrimentally affect the sensitivity of the detector due to limited dynamic range on the output stage. Our prior research in this area [27] shows that AP mines do not have a significant response at frequencies below a few kHz. A normalised magnitude of 0.5 has been used as a compromise such that this component may be useful for subsequent multi-spectral soil compensation algorithms, while not demanding a disproportionate amount of drive signal.

**Figure 4:**
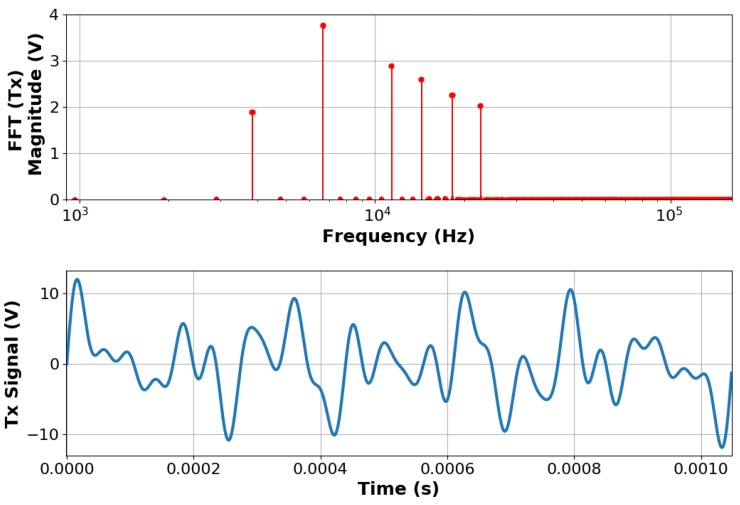
Transmit signal characteristics for MIS system.

The normalised transmit harmonics are used to calculate the time-domain signal. Each of these weighted harmonics is summed to create a composite waveform, which is subsequently scaled to optimise the dynamic range of the Digital-to-Analog converter on the MIS measurement system. This signal is then amplified to provide the signal shown in Figure 4.

### 2.2. Ground-Penetrating Radar

Ultra Wideband (UWB) GPR is a technology that has been developed for use in both humanitarian and military demining operations [16,34], and is generally considered to have the most potential amongst emerging detection technologies [8]. Daniels [34] gave an overview of existing GPR systems for landmine detection, including hand-held, vehicle based and airborne systems; the underlying mathematical models for soil and reflection loss have also been described in our previous work [35]. GPR technology has demonstrated that it is capable of detecting AP landmines but has not yet reached the stage where it can recognise and identify specific AP mine targets. To improve the overall detection performance and drive down the false alarm rate GPR technology will need to also gain better discrimination alongside the MIS capability. For these dual-technology systems, two well known systems that have made it to standard use are MINEHOUND and HSTAMIDS [8,34].

**Figure 5:**
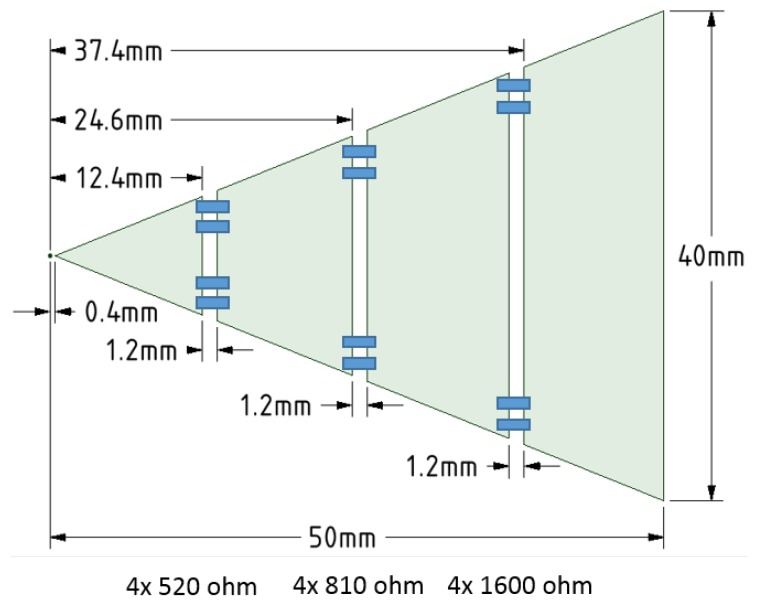
The geometry of one half of the loaded bowtie antennas.

**Figure 6:**
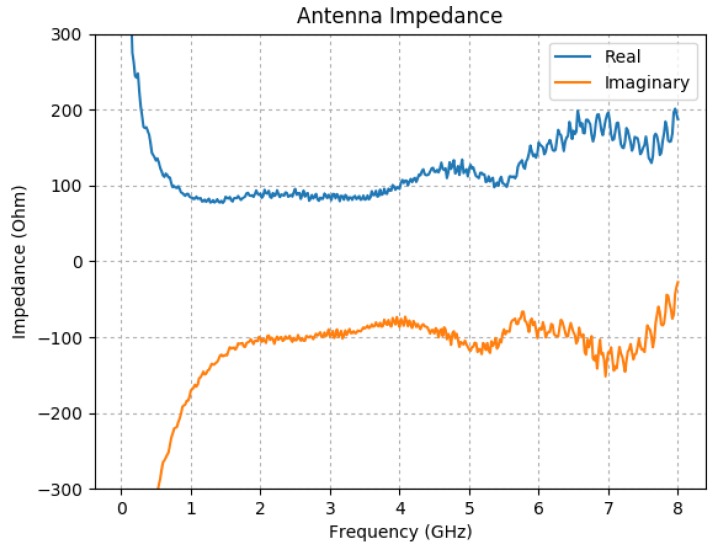
Antenna impedance of the loaded bowtie antenna.

The GPR system, at Technology Readiness Level 5, used in this paper uses the “stepped frequency continuous wave” (SFCW) modulation scheme. The RF signals are generated and captured by an Anritsu MS46122A Vector Network Analyser (VNA) [36]. The key parameters of the VNA configuration are presented in Table 2. The GPR system uses a pair of resistively loaded bowtie antennas; their geometry and loading profile is shown in Figure 5. The antennas are fed using tapered microstrip baluns based on the design by Vinayagamoorthy et al. [37], and are manufactured on Rogers RT Duroid 5880. The input impedance of the antennas was measured with a two-port VNA by using a twin semi-rigid line (TSR) as described in [38]; the result is shown in Figure 6. The antennas are separated by a septum, made using a bespoke carbon-loaded polymer sheet (50 Ω/sq.), to reduce the direct coupling. The GPR housing was filled with radar absorbing material (RAM) to reduce the unwanted radiation from the back and sidelobes. The septum and RAM filling were optimised to reduce the cross-talk between the antennas.

### 2.3. Dual Modality System

**Figure 7:**
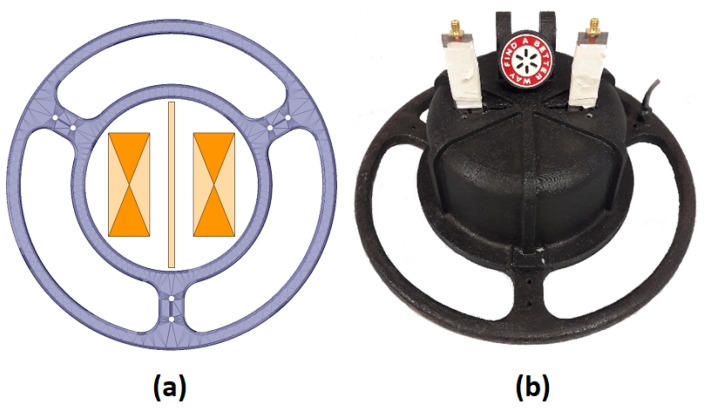
(**a**) The location of GPR antennas within the MIS sensor head; and (**b**) photograph of the combined sensor head.

**Figure 8:**
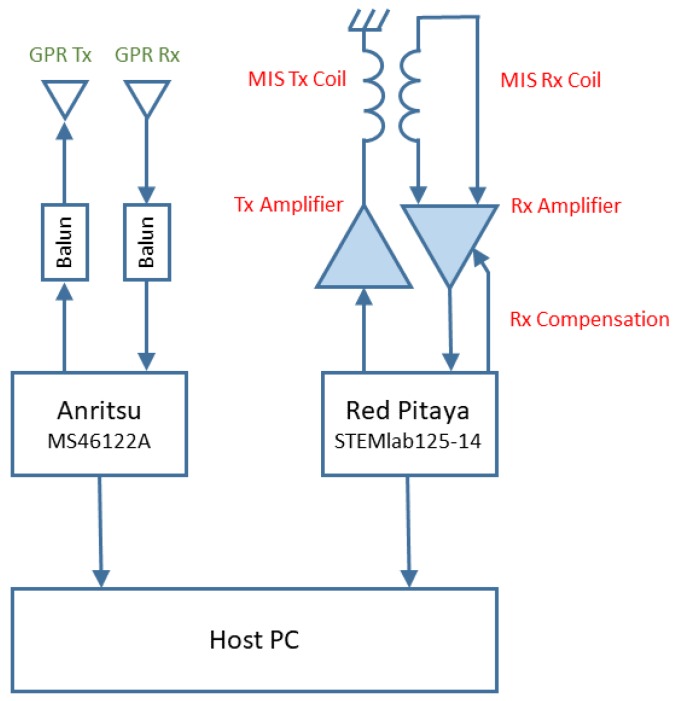
Schematic of combined MIS/GPR system.

The position of the GPR system antennas and septum within the MIS sensor head is shown on the left of Figure 7, with a photograph of the combined MIS/GPR sensor head on the right. A schematic diagram of the system is shown in Figure 8.

The integration between the two modalities places restrictions on both sensors. For example, the location of the GPR antennas inside the inner coil of the metal detector limits both their size and the maximum spacing between them. This rules out the use of many types of antennas. Furthermore, the presence of the metallic components in the GPR sensor inside the MIS coils could potentially upset the balancing between the MIS coils. The bowtie antenna was chosen for both its size and low metal content, and the slots (see Figure 5) across the antennas further limit the flow of eddy currents. The GPR housing does not rely on metallic shielding, but rather uses a combination of RAM and carbon loaded polymer. These measures are implemented to reduce the imbalance that is introduced between the MIS coils, and the resulting signal is small enough that it can be subtracted electronically.

## 3. Experimental Methodology

The results from a laboratory-based experiments are reported here. The relative permittivity of sand was measured using a Delta-T WET-2 sensor. The WET-2 sensor makes this measurement at a frequency in the tens of MHz rather than in the GPR range of 300 MHz to 6 GHz.

### 3.1. MIS Methodology

The MIS sensor is calibrated using a cylindrical ferrite sample (5 mm diameter by 16 mm length, type 4B1 Ferroxcube ferrite) located 7 cm away from the sensor. This ferrite sample is used as both a phase and a magnitude reference for the detector, and all measurement magnitudes in this paper are expressed in normalised units relative to this sample piece.

The signal from typical mineralised soils is predominantly present in the real part of the complex response of inductive metal detectors. The actual soil response cannot be considered as entirely real, but the imaginary component is significantly less sensitive to mineralised ground than the real component [39]. Consequently, to mitigate the effects of unwanted signals caused as a result of soil mineralisation, the analysis of MIS signals considered in this paper concentrates on the processing of the imaginary part of the spectroscopic signals. This removes the need for additional ground compensation algorithms. The comparison of real and imaginary components of spectroscopic metal detector data is further examined in Section 4.

The MIS responses were recorded at a rate of 60 Hz, and were augmented with positional information provided by a scanner system. The results presented are the product of a scattered interpolant function, which was used to convert the original dataset from scattered positions to a Cartesian grid.

### 3.2. GPR Methodology

The GPR system uses a VNA to form a SFCW radar which is used to capture a 3-D dataset of amplitude data. The configuration of the VNA is shown in Table 2. The measured amplitude data are processed to reconstruct the position of impedance discontinuities within the soil. A Stolt migration [40] method was used to perform this data reconstruction. The implementation used here is based on the descriptions found in [41,42]. Since the WET-2 sensor records the permittivity measurement at a much lower frequency than the VNA range, the sensor’s estimate is only used as the starting estimate for the GPR migration algorithm.

The GPR system cannot be directly calibrated at the input to the antennas due to the presence of the tapered microstrip baluns, whose balanced ends cannot be connected to a standard VNA calibration kit. Instead, a simplified calibration method was chosen whereby each frequency-domain measurement was divided, as a complex number, by a “through” measurement of the coaxial cables.

The GPR data were recorded at a rate of 15 Hz, and the resulting dataset was interpolated onto a Cartesian grid using a nearest-neighbour method.

### 3.3. Test Objects

**Figure 9:**
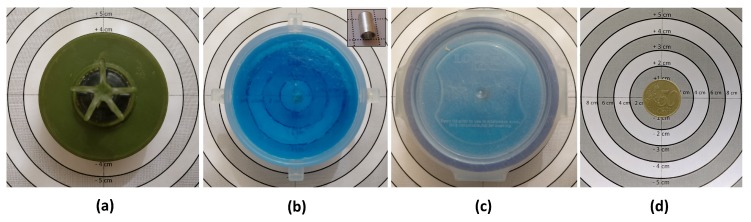
Test objects used: (**a**) PMA-2 surrogate; (**b**) type A72 surrogate—lid removed with metallic component (inset) on 1 cm grid; (**c**) PMA-3 surrogate; and (**d**) 50 Euro cent coin.

**Figure 10:**
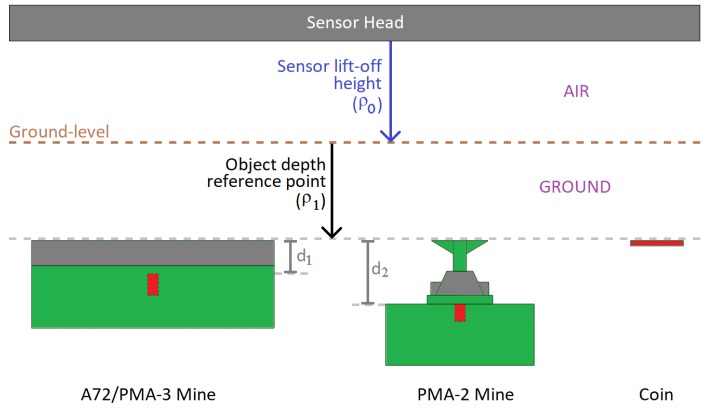
Description of object depth measurements and orientation with metallic components highlighted.

A total of four objects were used; these consisted of a 50 Euro cent coin, and three types of surrogate AP landmines: a PMA-2 surrogate, an A72 surrogate, and a PMA-3 surrogate. The PMA-2 surrogate was provided by Fenix Insight. Each of the landmine surrogates represented minimum-metal anti-personnel mines. The full list of objects and positions for the experiment is shown in Table 3 and the objects are shown in Figure 9. Each of these tables contains three values relating to depth and are defined with reference to Figure 10. The objects were buried such that their top was at a defined depth below ground level, $\rho_{1}$, however the observed depth by the MIS system refers to the total depth from the sensor to the metallic part of the objects. The depth of the metallic part of the mine from the detector also depends on the position of the metallic part within the mine and the offset height of the detector above ground (also known as “lift-off”). The internal positions of the metal components were difficult to measure in practice since the surrogate mines are resin-encapsulated, but the distances have been inferred to be $d_{1}\approx 15$ mm and $d_{2}\approx 30$ mm from [8,9], as shown in Figure 10. The total distance between the metallic reflector and the MIS sensor height was calculated by adding the lift-off, burial depth and internal metallic component location. This distance is shown in the last column of Table 3 and the depth of each object was measured according to the reference points shown in Figure 10.

### 3.4. Experimental Setup

**Figure 11:**
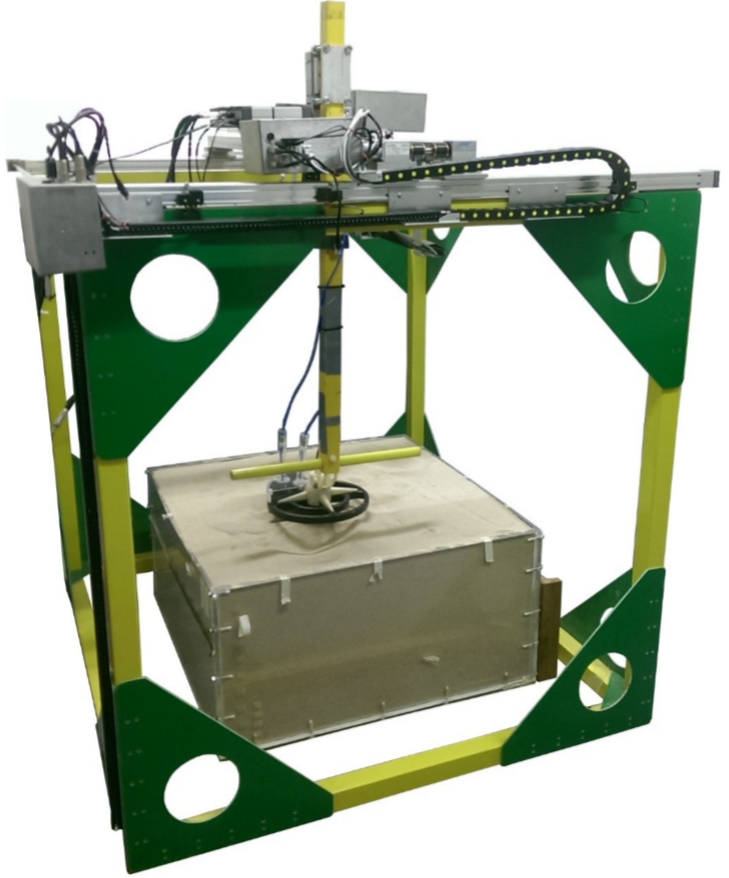
Three-dimensional scanner used for testing.

**Figure 12:**
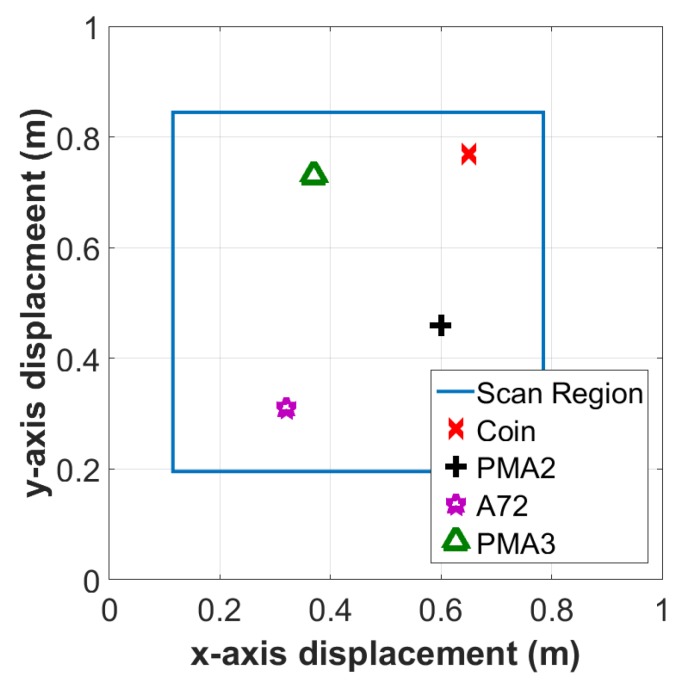
Locations for the objects buried in the sand.

This experiment was conducted in a laboratory with objects buried in a 1 m × 1 m × 0.5 m tank filled with dry sand. The size of the scan region is 0.68 m × 0.65 m. The sensor was moved by a mechanical x-y scanner (Figure 11) with a fixed distance to the surface of the sand. The relative permittivity of the sand was estimated by the WET-2 sensor to be $\varepsilon_{r}=2.6\pm 0.2$. The sand surface was flat (<5 mm variation) so the lift-off height is relatively constant at 2 cm ± 0.5 cm. Table 3 and Figure 12 show the position of the test objects within the tank.

## 4. Results and Discussion

### 4.1. Individual Sensors

**Figure 13:**
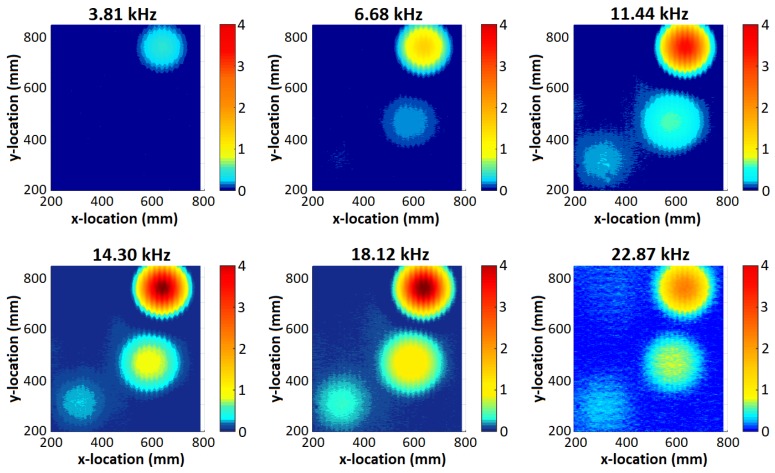
MIS test results—imaginary component.

**Figure 14:**
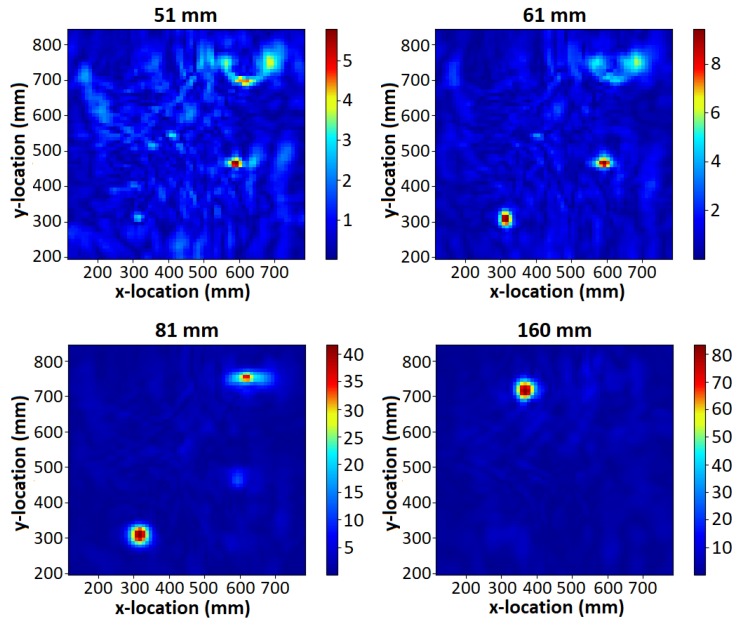
GPR testing results.

Figure 13 and Figure 14 demonstrate that all but the deepest target are clearly identifiable for the MIS system, and that the GPR identified all four targets. The depth of the PMA-3 surrogate (16 cm) was chosen to test the ability of the GPR system; it was not expected to be detectable by the MIS system given its minimum-metal content, and that it is buried much deeper than the system is expected to detect such targets. The MIS system has been designed according to typical demining procedures, which require clearance to a depth of 13 cm from original ground level [43,44]. The remaining three objects are all visible in the 18.12 kHz component, which provides the strongest response for the A72 surrogate. The A72 surrogate is not significantly detectable above the background noise, as shown in the colour map. The GPR is able to clearly identify the PMA-3 surrogate in the 160 mm C-scan slice; the remaining three items are visible in the 51 mm, 61 mm and 81 mm slices (see Figure 14). The depth of each object was estimated using B- and C-scans and the results are recorded in Table 4. The GPR gets a stronger return from the top of the base of the PMA-2 surrogate, rather than the top of its plunger. The recorded depth is therefore to the top of the base of the PMA-2 surrogate, which was measured to be 27 mm below the top of the plunger.

Table 4 shows the estimated location of detectable objects for the MIS and GPR sensors. These values, and all other positional estimates in this paper, are expressed to cm-level precision. This error is due to the step size of the measurement points—the y-axis resolution was 1 cm. Another source of error is the finite size of object, and the position of the maximum measurement response may not coincide with the centre of the object. However, with the exception of the GPR estimate for the location of the coin, the positional error is within 20 mm, which is close to the expected margin of error in burying of the objects (≤10 mm).

The MIS system shows almost no signal in areas in which the objects are not present. This is representative of the fact that the sand tank is a known metal-free area. For the GPR, the upper slices show a reduced signal-to-noise ratio (SNR) as a result of a combination of factors. Firstly, the system is susceptible to undulations in the surface of the sand. In addition, the magnitude of the response of the object in these slices close to the surface is almost an order of magnitude smaller than the deepest slice, and consequently the effect of noise from clutter in the image is more apparent than for deeper targets. The smaller response can be explained in part by the shape of the buried object, which is a PMA-2 surrogate in this case; the plunger at the top of the surrogate mine has a small surface area, leading to a smaller reflection. In depth slices located further away from the air–ground interface, the background clutter level drops and the targets stand out clearly.

### 4.2. Sensor Fusion

An important part of any dual-modality landmine detector system is how it handles the information coming from the two component sensors. This information can be fused to generate a single output from the detector based on underlying data from both modalities. Sensor fusion can be performed at different stages of the data acquisition and processing process. This paper explores feature-level sensor fusion, where each component sensor extracts features from the raw data, and the fusion algorithm makes a joint declaration based on all features from all sensors.

The starting point is to generate one feature each from both the MIS ($c_{md}\left(\vec{p}\right)$) and GPR ($c_{gpr}\left(\vec{p}\right)$) systems, corresponding to the confidence that an object is present at the point $\vec{p}$. These confidence values have been scaled such that they lie in the range 0≤c≤1. A third feature is extracted by the GPR sensor, corresponding to the depth of a buried objects. The fusion algorithms use these features to arrive at a target declaration $o\left(\vec{p}\right)\in \left\{0,1\right\}$ at point $\vec{p}$, where 0 indicates no target present and 1 indicates that a target is present.

(1) $$o\left(\vec{p}\right)=\left\{\begin{array}{cc} 1 & c_{gpr}\left(\vec{p}\right)\geq t_{gpr}\left(c_{md},d_{gpr}\right)\wedge c_{md}\left(\vec{p}\right)\geq t_{md}\left(c_{gpr},d_{gpr}\right)\\ 0 & c_{gpr}\left(\vec{p}\right)<t_{gpr}\left(c_{md},d_{gpr}\right)\vee c_{md}\left(\vec{p}\right)<t_{md}\left(c_{gpr},d_{gpr}\right) \end{array}\right.$$

In this feature-level fusion algorithm, $c_{md}$ and $c_{gpr}$ are compared against a threshold, which could be dependent on the other features. A positive detection is declared if both confidence values exceed their respective thresholds. The values of the thresholds determine the ultimate receiver operating characteristic (ROC) curve of the system. This fusion algorithm is described mathematically in Equation (1).

To achieve the best results, it is important to consider all three features simultaneously when coming to a joint target declaration. This is elaborated in the following section, demonstrating how all the features are used to positively identify every target.

**Figure 15:**
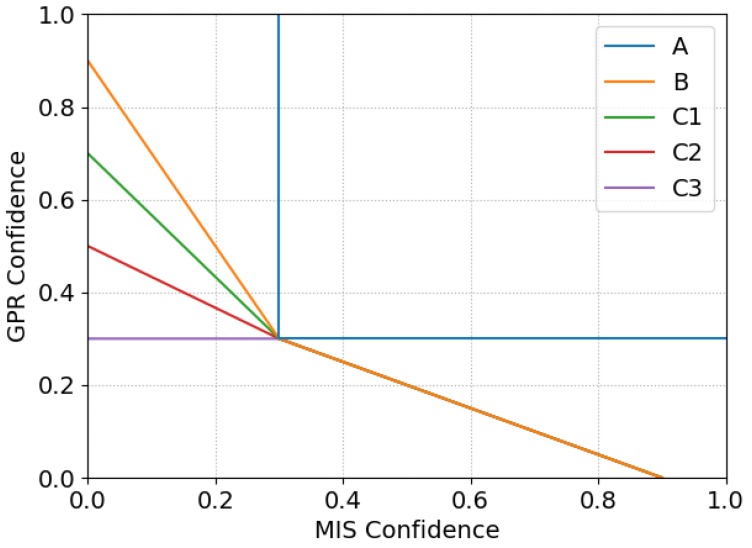
Decision surfaces showing how targets are classified based on the confidence from the two sensors, based on varying thresholds.

**Figure 16:**
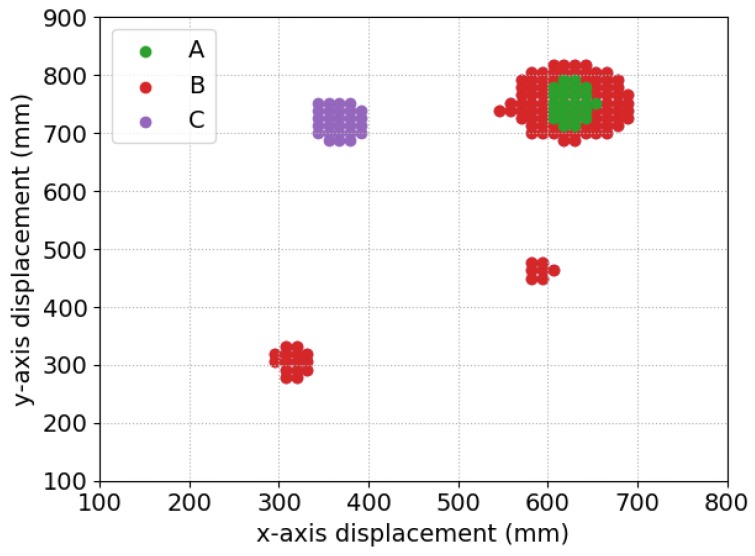
Detections based on the three different threshold calculations.

A simplified case of the algorithm in Equation (1) would consider the thresholds to be fixed values: $t_{gpr}=t_{md}=0.3$. The decision surface for this approach is drawn as line “A” in Figure 15. The pair ($c_{md}\left(\vec{p},c_{gpr}\left(\vec{p}\right)\right)$ can be seen as a point on this surface. Any points to above and to the right of line “A” lead to a positive detection. The output from the detection algorithm is shown in Figure 16, labelled as “A”.

Using these fixed threshold values, there is only one positive detection, on the coin. For every other object, the confidence from one sensor is too low, even if it is high for the other sensor. The values of $t_{gpr}$ and $t_{md}$ could be lowered to improve the sensitivity to these objects, but this would come at a cost of higher rates of false alarms. Based on these observations, an alternative approach can be implemented.

If one sensor reports very high confidence regarding the presence of an object, it is not necessary to require a high degree of confidence from the other sensor. This can be implemented by making the threshold for the MIS sensor dependent on the threshold of the GPR sensor and vice versa. Mathematically, this can be represented as follows.

$t_{gpr}\left(c_{md}\right)=t_{md}\left(c_{gpr}\right)$, shown below as t(c):

(2) $$t\left(c\right)=\left\{\begin{array}{cc} 0.3 & \mathrm{if}\;c<0.3\\ 0.3-\frac{c-0.3}{2} & \mathrm{if}\;0.3\leq c<0.9\\ 0.0 & \mathrm{if}\;c\geq 0.9 \end{array}\right.$$

Line “B” in Figure 15 shows how the threshold for one sensor is gradually reduced as the confidence value from the other sensor increases. This approach improves the results of the detection algorithm such that it positively detects three out of the four objects. The additional detections from this approach are labelled as “B” in Figure 16.

The second algorithm detects all objects except for the PMA-3 surrogate landmine buried at 16 cm depth. As explained previously, it is not expected that the MIS system will be able to detect such small objects at such depths. Therefore, the algorithm could be further improved by taking into account the depth of the objects detected by the GPR. If this depth is beyond the detection range of the MIS system, the threshold for the MIS system can be reduced.

**Figure 17:**
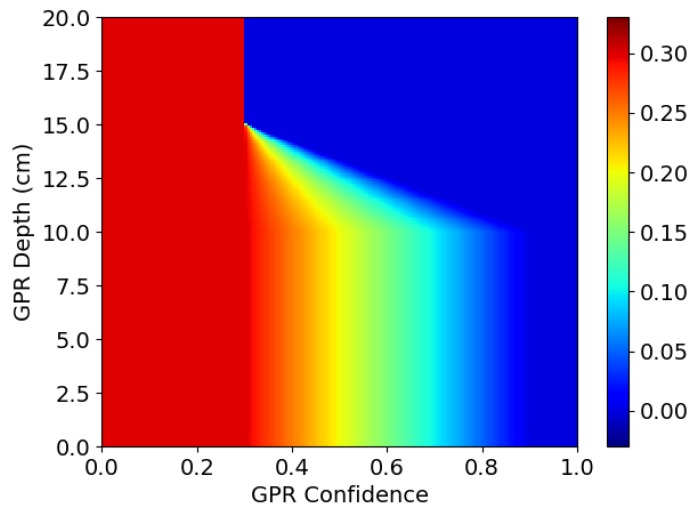
MIS detection threshold function as function of object depth (as reported by GPR) and GPR confidence.

In the third algorithm, the depth of the object ($d_{gpr}$) is extracted as an additional feature from the raw GPR data. The detection thresholds now become two-dimensional functions, with $t_{gpr}\left(c_{md},d_{gpr}\right)=tgpr\left(c_{md}\right)$ as in Equation (2) and $t_{md}\left(c_{gpr},d_{gpr}\right)$ as shown in Figure 17.

The lines “B”, “C1”, “C2” and “C3” in Figure 15 show how the decision surface changes as a function of depth. At 10 cm depth, the decision surface is unchanged from before, following line “B”. As the depth increases to 11.7 cm, the decision surface changes to follow line “C1” and, at 13.3 cm, it follows line “C2”. Finally, for depths of 15 cm and greater, it follows line “C3”.

Figure 16 shows the additional detections from this algorithm labelled as “C”. This algorithm, using all features from the sensors, detects all four buried objects.

### 4.3. Multi-Modal Data Visualisation

**Figure 18:**
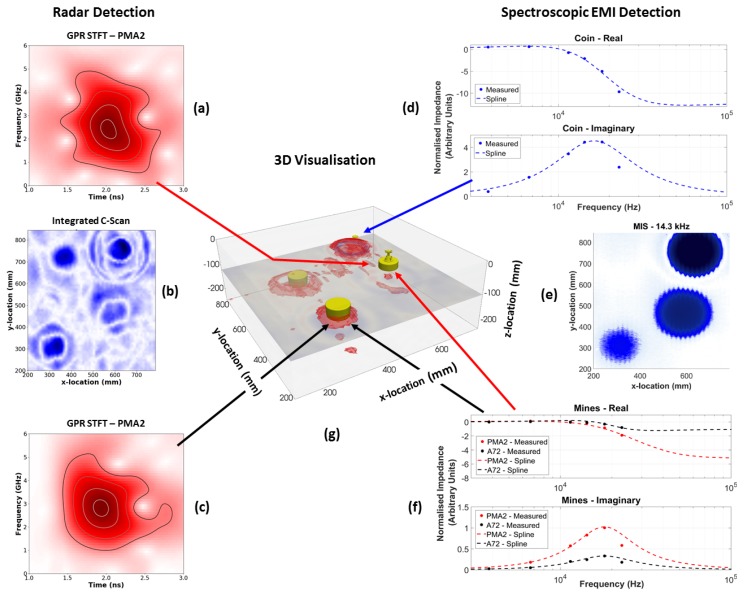
Multi-modal dataset: (**a**) GPR spectral analysis for PMA-2; (**b**) Integrated C-Scan of GPR data; (**c**) GPR spectral analysis of A72; (**d**) MIS analysis of coin; (**e**) MIS spatial analysis of test area at 14.3 kHz; (**f**) MIS analysis of mine targets; and (**g**) test object locations with GPR perturbations overlaid.

Figure 18 shows a composite image containing the captured MIS and GPR spectral information for each target in this experiment; Figure 18a–c shows GPR data, and Figure 18d–f shows MIS data. Figure 18g shows the position of the target objects with GPR information overlaid, visualised in 3D using ParaView [45]. This information takes the form of a cut-plane through the GPR data cube at z=−120 mm, with additional isosurfaces resulting from thresholding the amplitude of the GPR response (in red). The latter is represented using red highlights in the figure, and it is possible to see that these highlights correspond with the known object locations.

The GPR plot shown in Figure 18b is an integrated C-scan between range values. In this image, the C-scans from the different depths where the targets were buried have been summed together to generate a single, top-down view which contains the responses from every target. This figure shows all four buried targets, but the detection of the PMA-2 surrogate is very marginal, similar to the results presented in Figure 14. No migration algorithms were applied to the data in this figure. The images in Figure 18a,c show the result of a Short-Time Fourier Transform (STFT) of the A-scan over a target. These plots show the spectral content of the signal as a function of time, with the contour lines showing the 25%, 50%, 75% and 95% amplitude levels. It has been shown before that the spectral content of the target response can be used to improve target discrimination [46]. These figures demonstrate the richness of the data collected by the prototype dual-modality sensor and the possibilities for future developments regarding discrimination and classification algorithms.

The MIS spectral plots shown in Figure 18d,f can be compared with those shown in Figure 2. A spline function has also been plotted on these figures to aid interpretation of the data. It can be observed that each object has a discernible spectroscopic response, with variations in the magnitude of the real and imaginary components, the final asymptotic value of the real components, and the frequency of the peak value of the imaginary components. It can be seen that the 22.87 kHz component of all three objects shows a consistent error compared to the spline function. The SNR of the detector is poorest at this frequency (as also shown in Figure 13), and consequently it is believed that there may have been some phase noise of the order of 7${}^{\circ }$ on the calibration signal used to reference the detector. For all other frequency components, it can be seen that the captured data follow the anticipated trend. Figure 18e shows the spatial MIS response at a single frequency to the coin, A72, and PMA-2, the locations are directly comparable with that in Figure 18b,g.

## 5. Conclusions

The results reported here show that it is possible to spatially locate metallic clutter items and minimum-metal mine surrogates to within 20 mm of their known positions. When tested in sand, the ground penetrating radar was able to detect all targets, and the metal detector all but the deepest target. The depth of the deepest target was considered to be below the typical operating depth of metal detector systems.

However, the metal detector performance requires improvement in order to detect minimum-metal targets with metal components in the range of 10–15 cm depth from the sensor head (the case for the A72 surrogate in these experiments). Some care must be taken when interpreting the GPR data for shallow targets, as this is where the signal-to-clutter ratio has been observed to be poorest. Further improvements to the antenna design are necessary to alleviate this problem.

In this work, the two separate sensor systems were integrated on the same sensor head. The MIS and GPR sensors shared a common power supply and host PC, were mechanically integrated on the same sensor head, and operated simultaneously. An example sensor fusion algorithm was investigated, based on feature-level fusion. The effects of different ways of calculating the threshold values for detection were shown. The added value of combining GPR and MIS sensors was shown by extracting the estimated depth of the objects and including this feature in the fusion algorithm.

Both detection modalities are able to operate effectively when integrated onto the same sensor head, and are operated simultaneously. This paper shows the potential for improving performance of landmine detection systems by combining a magnetic induction spectroscopy system with a ground penetrating radar to enable automated object location. It also demonstrates the richness of the combined MIS/GPR dataset that the system is able to measure, which allows for the potential of expanding the algorithm to implement object classification in the future.

## Figures and Tables

**Table 1:** MIS transmit signal characteristics.

Harmonic	Frequency	Normalised Magnitude	RMS Magnitude
Index		(Arbitrary Units)	(A·Turns)
4	3.81 kHz	0.50	2.07
7	6.68 kHz	1.00	2.34
12	11.44 kHz	0.77	1.06
15	14.30 kHz	0.69	0.78
19	18.12 kHz	0.60	0.53
24	22.87 kHz	0.54	0.37

**Table 2:** Summary of the key VNA parameters used.

Number of Steps	220
Frequency step size	27 MHz
Start frequency	270 MHz
Stop frequency	6183 MHz
I.F. bandwidth	1 kHz
TX Power	−3 dBm

**Table 3:** Location of metallic component of target objects used in the experiment.

Object Name	Location	Depth	Lift-Off	MIS Depth
	(x,y) (m)	$\rho_{1}$ (cm)	$\rho_{0}$ (cm)	(cm)
50 cent coin	(0.65,0.77)	8	2	10
PMA-2 surrogate	(0.60,0.46)	2	2	7
A72 surrogate	(0.32,0.31)	6.5	2	10
PMA-3 surrogate	(0.37,0.73)	16	2	19.5

**Table 4:** Estimated positions of the objects in the experiment.

Modality	Object	Known (x, y ,z)	Estimated (x, y, z)
	Name	(m)	(m)
MIS	Coin	(0.65,0.77,0.080)	(0.64,0.76,−)
MIS	PMA-2	(0.60,0.46,0.047)	(0.59,0.46,−)
MIS	A72	(0.32,0.31,0.065)	(0.32,0.31,−)
MIS	PMA-3	(0.37,0.73,0.160)	(−,−,−)
GPR	Coin	(0.65,0.77,0.080)	(0.62,0.75,0.051)
GPR	PMA-2	(0.60,0.46,0.047)	(0.59,0.47,0.061)
GPR	A72	(0.32,0.31,0.065)	(0.32,0.31,0.081)
GPR	PMA-3	(0.37,0.73,0.160)	(0.37,0.73,0.152)
